# Subterranean Fire in Belgium

**Published:** 1859-12

**Authors:** 


*Subterranean Fire in Belgium.*—The *Union*, of Charlero, in Belgium, says: "For some days, and especially for some nights past, flames of a bluish color are to be seen in the neighborhood of the village of Falisole, and they leave after them a strong sulphurous smell. They come from the bed of coal called the Grande Masse, which has been burning underground for about 40 years, in spite of all efforts to extinguish or check the conflagration. The soil above the Grande Masse contains numerous crevices, from which puffs of heat often come; the crops on it ripen two or three weeks sooner than on other land, and in winter the snow always melts; whilst from an abandoned well, smoke continually arises. This subterranean fire, the only one which exists in Belgium, is said to have been wilfully occasioned by some of the inhabitants of Falisole, as an act of spite to other inhabitants of the village for having extracted more coal than the former thought right—the pit being common property."—*Medical and Surgical Journal.*

